# Normal regional pulse wave velocity predicts absence of aortic luminal growth in patients with Marfan syndrome: a comprehensive MRI-study

**DOI:** 10.1186/1532-429X-14-S1-P131

**Published:** 2012-02-01

**Authors:** Eleanore Kroner, Arthur Scholte, Patrick Koning, Pieter J van den Boogaard, Rob J van der Geest, Lucia J M Kroft, Hildo J Lamb, Yvonne Hilhorst-Hofstee, Maarten Groenink, Teodora Radonic, Barbara J Mulder, Ernst E van der Wall, Jeroen J Bax, Albert de Roos, Johan H Reiber, Jos J Westenberg

**Affiliations:** 1 Radiology, Leiden University Medical Center, Leiden, Netherlands; 2 Cardiology, Leiden University Medical Center, Leiden, Netherlands; 3 Cardiology, Academic Medical Center, Amsterdam, Netherlands; 4 Clinical Genetics, Leiden University Medical Center, Leiden, Netherlands

## Summary

By using a comprehensive MRI-approach in patients with Marfan syndrome, increased regional pulse wave velocity in the ascending aorta can predict in 42% of the cases aortic luminal growth in the ascending aorta, while normal PWV can predict in 89% absence of luminal growth.

## Background

The leading cause of premature death in patients with Marfan syndrome (MFS) is aortic dissection after progressive dilatation due to local increased wall stiffness, occurring most prominently in the ascending aorta. Aortic pulse wave velocity (PWV) is a marker of wall stiffness [Nollen, et al. Eur Heart J 2004]. Regional PWV can be accurately determined from in-plane multi-directional velocity-encoded (VE) MRI [Westenberg, et al. JMRI 2010]. The study objective was to test whether regional PWV can predict regional aorta dilatation at 2-year follow-up (FU) in MFS.

## Methods

In twenty-one MFS patients (mean age 36±15 years, 11 male) regional PWV and aortic luminal areas were assessed by 1.5T MRI (Philips, The Netherlands). At 2-year FU, the incidence of luminal growth was determined.

**Figure 1:**
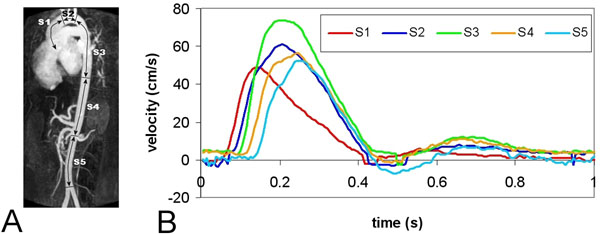
(A) Contrast-enhanced MRA of the aorta of a patient with Marfan syndrome. Five aortic segments are defined: ascending aorta (S1), aortic arch (S2), thoracic descending aorta (S3) suprarenal abdominal aorta (S4) and subrenal abdominal aorta (S5). Per segment, mean luminal area is defined and compared at 2-year follow-up. (B) Pulse wave propagation between the five segments defines Pulse Wave Velocity.

In detail, a contrast-enhanced MR angiogram of the full aorta was obtained by first-pass imaging of a 25mL contrast bolus Dotarem (0.5mmol/mL, infusion rate 2mL/s), with 3D T1-weighted gradient-echo. Image analysis was performed using in-house developed software with automated centerline detection [de Koning et al., MRM 2003] and 3D deformable modeling [Makowski et al., LNCS 4072 2006]. Cross-sectional luminal areas were measured at 200 equidistantly-spaced sample points along the centerline. The aorta was divided into five segments (Figure 1A): ascending aorta (S1), aortic arch (S2), thoracic descending aorta (S3), suprarenal abdominal aorta (S4) and infrarenal abdominal aorta (S5).

Next, regional PWV was obtained for each segment by wave propagation analysis (Figure 1B) from multi-slice two-directional velocity-encoded MRI, covering the full aorta by three double-oblique sagittal slices with velocity sensitivity 150 cm/s in anterior-posterior and feet-head (in-plane) direction. Regional PWV was calculated by the length of each segment and the transit-time of the flow velocity wave propagating through this segment [Westenberg et al., JMRI 2010]. Regional PWV was compared with age-related normal values [Westenberg et al., JMRI 2011] and considered increased when exceeding age-related PWV by two standard errors.

For twenty-one patients, regional PWV at baseline was compared with cross-sectional luminal area growth from baseline to FU. A mean luminal area increase >14.6mm2 (i.e., two voxels) was considered significant growth.

## Results

In twenty-one MFS patients, a mean aorta trajectory of 44±4cm was evaluated. Significant luminal growth at FU was reported in 47 out of 105 aortic segments (45%) and regional PWV at baseline was increased in 42 segments. The incidence of luminal growth per segment and the sensitivity and specificity for PWV predicting luminal growth are presented in Table 1.

**Table 1:** predictive performance of regional PWV-assessment on luminal growth at 2-year follow-up

Aortic region	Incidence of luminal increase	Sensitivity	Specificity
S1	12	42%	89%
S2	15	27%	83%
S3	11	27%	70%
S4	7	29%	36%
S5	2	100%	35%

## Conclusions

Increased regional PWV in the ascending aorta in MFS patients predicts in 42% of the cases aortic luminal growth, while normal PWV predicts in 89% absence of luminal growth. Specificity decreases for more distal segments.

## Funding

Netherlands Heart Foundation (Project 2006B138).

